# Editorial: FEAST of biosensors: food, environmental, and agricultural sensing techniques

**DOI:** 10.3389/fbioe.2024.1482905

**Published:** 2024-09-10

**Authors:** Kang Mao, Hua Zhang, Zhugen Yang

**Affiliations:** ^1^ State Key Laboratory of Environmental Geochemistry, Institute of Geochemistry, Chinese Academy of Sciences, Guiyang, China; ^2^ Cranfield University, Cranfield, United Kingdom

**Keywords:** biosensor, environmental analysis, agricultural analysis, food safety, health

Biosensors are analytical devices that involve biological elements, such as nucleic acids, antibodies, enzymes, cells and microorganisms with a physicochemical (such as optical, electrical and mechanical) detectors. In the last 5 years the number of publications with the keyword “biosensor” is over 5,000 per year from the Web of Science and various biosensors are being designed and fabricated for high-efficiency, multiplex-functionality and high-flexibility sensing applications in the field of food, environment and agriculture. Many existing biosensors have the inherent capacity to achieve such goals; however, they require further development into and make it more easy-to-use and reduce cost without compromising the analytical performance such as sensitivity and specificity, as well as at a greater scale than heretofore possible.

In this Research Topic, we have overall gathered four research articles and discussed how biosensors have great, as yet unmet, promise to provide widespread and potentially low-cost monitoring of chemicals, such as metronidazole, and microbes, such as Ralstonia solanacearum, in food, environmental and agricultural application. We briefly outlined these works in this special as follows:

Estrone, as an endogenous estrogen, has a variety of physiological functions in human body, while it is a widely distributed and highly disturbing environmental endocrine disruptor in water. Cao and Chen developed a stable and reproducible molecularly imprinted membrane-electrochemical luminescence (MIP-ECL) sensor for ultra-sensitive and accurate quantitative detection of estrone. The Ru (bpy)_3_^2+^/multi-walled carbon nanotubes/Nafion/gold electrodes prepared by surface electrostatic adsorption and ion exchange were modified with MIP synthesized by sol-gel method with the capability to recognize estrone to form a sensor, which simultaneously possesses ECL’s advantage of high sensitivity and MIP’s advantage of high selectivity. Moreover, the addition of carboxylated multi-walled carbon nanotubes improved the functionalization of the gold electrode surface and increased the binding sites of MIP. Meanwhile, the good conductivity of multi-walled carbon nanotubes promoted electron transfer and further improved the sensitivity of the sensor. The sensor showed a wide linear interval ranging from 0.1 μg/L to 200 μg/L with the detection limit of 0.0047 μg/L, which indicates that the quantitative results obtained by this sensor are accurate and can be used for rapid *in situ* determination of estrone in practical samples.

Excessive residue of metronidazole in food is harmful to the human body and herein development of a portable tool for metronidazole detection on-site is an urgent demand. Zhang et al. constructed a smartphone colorimetric biosensor for the detection of metronidazole in milk by using the newly identified mutated aptamer combined with the smartphone dark box. The principle of color change is caused by the aggregation state of AuNPs. Smartphones act as reading instruments. The detection can be completed in just a few seconds without the aid of instruments, achieving a range of 6.7–44.4 μM and a detection limit of 0.15 μM and, demonstrating the important applications in food detection.

Bacterial wilt caused by the aerobic, Gram-negative pathogenic species *Ralstonia solanacearum* is a major disease impacting commercial agriculture worldwide. An urgent priority in control of bacterial wilt is development of rapid, sensitive, effective methods for detection of Ralstonia solanacearum. Fan et al. fabricated a novel nucleic acid biosensor with high sensitivity and strong specificity by the combination of loop-mediated isothermal amplification (LAMP) with CRISPR/Cas12a assay for detection of Ralstonia solanacearum in tomato through two visual detection techniques, involving naked-eye observation of fluorescence and lateral flow strips. The LAMP/Cas12a assay accurately detected Ralstonia solanacearum phylotype I in 14 test strains and the overall detection process took less than 2 h and did not require professional lab equipment. Ralstonia solanacearum in tomato stem tissue and soil samples from two field sites with suspected Bacterial wilt infection was identified accurately, suggesting that LAMP/Cas12a assay can be developed as an effective, inexpensive technique for field detection and monitoring of Ralstonia solanacearum.

Microelectrode arrays are extensively utilized in encoding studies of retinal ganglion cells, while conventional planar microelectrode arrays face limitations in studying retinal ganglion cells due to poor coupling between electrodes and retinal ganglion cells, resulting in low signal-to-noise ratio and limited recording sensitivity. To overcome these challenges, Zhang et al. employed photolithography and electroplating processes to fabricate a 3D microelectrode array based on the planar microelectrode arrays platform and explored retinal ganglion cells encoding to multi-modal stimulation using 3D microelectrodes arrays, which exhibited several improvements, including lower impedance and phase delay, as well as higher charge storage capacity. Leveraging the advanced 3D microelectrode arrays, they investigated the encoding characteristics of retinal ganglion cells under multi-modal stimulation and found that electrical stimulation elicited more effective retinal ganglion cells firing, while optical stimulation enhanced retinal ganglion cells synchrony. These findings hold promise for advancing the field of neural encoding.

Although there are only four articles, we can see the flourishing development and unique advantages of sensing techniques in the field of food, environmental, and agricultural applications. With the emergence of emerging fields, such as wastewater-based epidemiology (Mao et al., 2021), the application of sensors will be further expanded; Meanwhile, the emergence of some new techniques, like machine learning (Simon et al., 2024), will optimize and improve analytical performance of sensor itself. All these will lead to a grand FEAST of biosensors soon.
